# Spatiotemporal clustering and Random Forest models to identify risk factors of African swine fever outbreak in Romania in 2018–2019

**DOI:** 10.1038/s41598-021-81329-x

**Published:** 2021-01-22

**Authors:** Mathieu Andraud, Stéphanie Bougeard, Theodora Chesnoiu, Nicolas Rose

**Affiliations:** 1 Epidemiology, Health and Welfare Research Unit, Ploufragan-Plouzané-Niort Laboratory, Anses, French Agency for Food, Environmental and Occupational Health & Safety, 22440 Ploufragan, France; 2 ANSVSA (Autoritatea Națională Sanitară Veterinară și pentru Siguranța Alimentelor), Piaţa Presei Libere nr. 1, Corp D1, Sector 1, 013701 Bucharest, Romania

**Keywords:** Computational models, Data acquisition, Data processing, Machine learning, Computational science, Risk factors

## Abstract

African swine fever (ASF) has affected Romania since July 2017, with considerable economic and social consequences, despite the implementation of control measures mainly based on stamping out of infected pig populations. On the basis of the 2973 cumulative recorded cases up to September 2019 among wild boars and domestic pigs, analysis of the epidemiological characteristics could help to identify the factors favoring the persistence and spread of ASF. A statistical framework, based on a random forest methodology, was therefore developed to assess the spatiotemporal features of the epidemics and their relationships with environmental, human, and agricultural factors. The landscape of Romania was associated with the infection dynamics, particularly concerning forested and wetland areas. Waterways were also identified as a pivotal factor, raising questions about possible waterborne transmission since these waterways are often used as a water supply for backyard holdings. However, human activity was clearly identified as the main risk factor for the spread of ASF. Although the situation in Romania cannot be directly transposed to intensive pig farming countries, the findings of this study highlight the need for strict biosecurity measures on farms, and during transportation, to avoid ASF transmission at large geographic and temporal scales.

## Introduction

African swine fever virus (ASFV) is a DNA virus belonging to the Asfarviridae family^1^ and displays considerable genetic diversity. The virus can infect wild and domestic Suidae. Certain strains are associated with a case-fatality rate close to 100%, especially in domestic pigs which are highly sensitive to the virus. There is currently no vaccine available to control the infection. As a result, African swine fever (ASF) has considerable economic consequences not only at the herd level, but also at the country level, due to the ban on exports following its emergence in a country officially free from the disease^2,3^.

Sub-Saharan Africa has been seriously affected by ASF, with the virus being endemic in several countries^4^. The virus was introduced into Europe several times in 1957 and 1960 (Portugal), and has since been introduced occasionally into several European countries with sporadic cases that were rapidly controlled. However, eradication from the Iberian Peninsula was only achieved in 1995. In 1978, the virus was also introduced into Sardinia, where it is still endemic today. Continental Europe was therefore considered free from ASF until 2007, when the virus was introduced into Georgia through infected waste coming from a ship originating from Eastern Africa, with the waste being fed to domestic backyard pigs^5^. The virus, of genotype 2, is highly virulent with a case-fatality rate higher than 95% in pigs and wild boars. It spread through Russia and Ukraine, where it affected in particular wild boars but also domestic pigs, especially those reared in backyards with poor biosecurity measures^6–8^. The spread of ASFV extended to Eastern Europe, the Baltic countries, before emerging in Poland^9^. The first ASF case in the Czech Republic was recorded in June 2017. In late February 2019, after 6 months without any ASF-positive cases owing to strong control measures, the country was declared ASF-free. In these Eastern European countries, the main population affected was wild boars, with only sporadic cases in domestic pigs due to failure of biosecurity, and high infection pressure in the wild reservoir^10^.

In Romania, the first notification was reported in July 2017 in the north-west area of Satu Mare. This initial incursion had limited consequences in terms of local spread^11^. However, an ongoing epidemic was initiated 1 year later, with more than 1000 cases reported up to October 2018 among both domestic pigs and wild boars. The incidence of outbreaks on domestic pig farms was particularly striking in summer 2018 with more than 650 new outbreaks in 2 months between June 10 and August 19, 2018. By summer 2019, the cumulative number of cases in Romania reached nearly 3000, with 13,665 pig owners compensated, at a total cost of 70 million Euros (http://www.ansvsa.ro/blog/actualizarea-situatiei-privind-evolutia-pestei-porcine-africane-42/).

The situation concerning ASF in Romania is unique due to both the duration of the outbreak and the number of cases that were reported in the domestic pig compartment. In view of the economic and social consequences of ASF outbreaks, there is a strong and urgent need to understand and explore the drivers of infection spread, to gain insights from these disastrous events. Existing studies have already provided estimates for transmission parameters from field and experimental data^7,12–16^. We aim here to unravel the determinants of ASF spread at the national and local scales in Romania. Two approaches were adopted. On the one hand, a spatiotemporal analysis was carried out to identify clusters, providing insights on the epidemiologic relationships between the different outbreaks. On the other, topographic and structural determinants of the transmission patterns were identified through a risk factor analysis, allowing us to assess the risk of ASF expansion to non-infected areas.

## Results

### Data description

**Figure 1:**
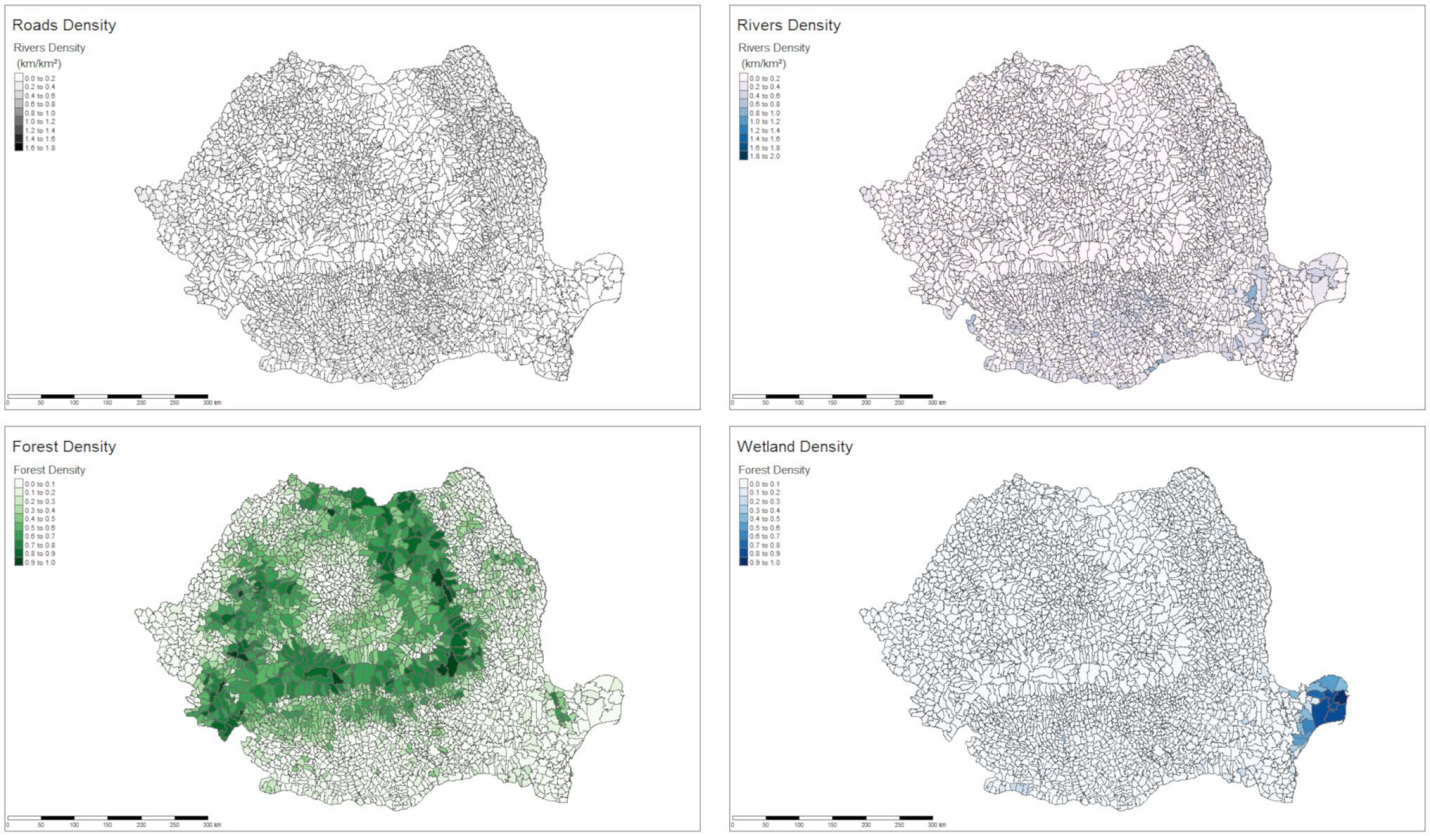
Cartography of the geographic variables. All data are presented as densities at the commune level.

Romania covers a total area of 238,439 km^2^ and is divided into 42 counties and 2939 communes with a very wide range of areas from 1.1 to 797 km^2^. The smallest commune is the town of Victoria in Brasov County and is surrounded by the commune of Ucea de Jos, while the largest is in the Danube Delta Nature Reserve in Tulcea county (Murighiol). Geographic, hydrologic, and transportation infrastructure data were analyzed at the commune level and are therefore discussed as densities. According to EuroglobalMap specifications, 40,000 km of roads, forming a logical transport network at a map scale of 1:1,000,000 are present across the country, with densities ranging between 0 and 1.7 km/km^2^ at the communal scale. The regions Danube Delta and Carpathian mountains show the lowest road densities. Railways are relatively sparsely representing 8000 km of line length, mainly located in the south and converging towards Bucharest, and in the northwestern part of the country. Wetlands and lakes are quasi-exclusively located in the Danube Delta Nature Reserve, covering between 55 and 95% of the communes in this area. The Carpathian Mountains represent the major forestry region, although wooded areas are also present at lower altitudes, especially in the county of Braila where the Danube flows. Waterways mostly originate in the Carpathian Mountains and flow into the Danube then to the Black Sea, forming natural borders with Bulgaria to the south, and Ukraine to the north along the Danube Delta. These data are illustrated in Fig. 1 (interactive view in html version).

The average population density was evaluated at 94 inhabitants/km^2^, with high density values in cities (7700 inhabitants/km^2^ in Bucharest), and some areas with densities close to 0 (Danube Delta Nature Reserve, or high-altitude mountains).

**Figure 2:**
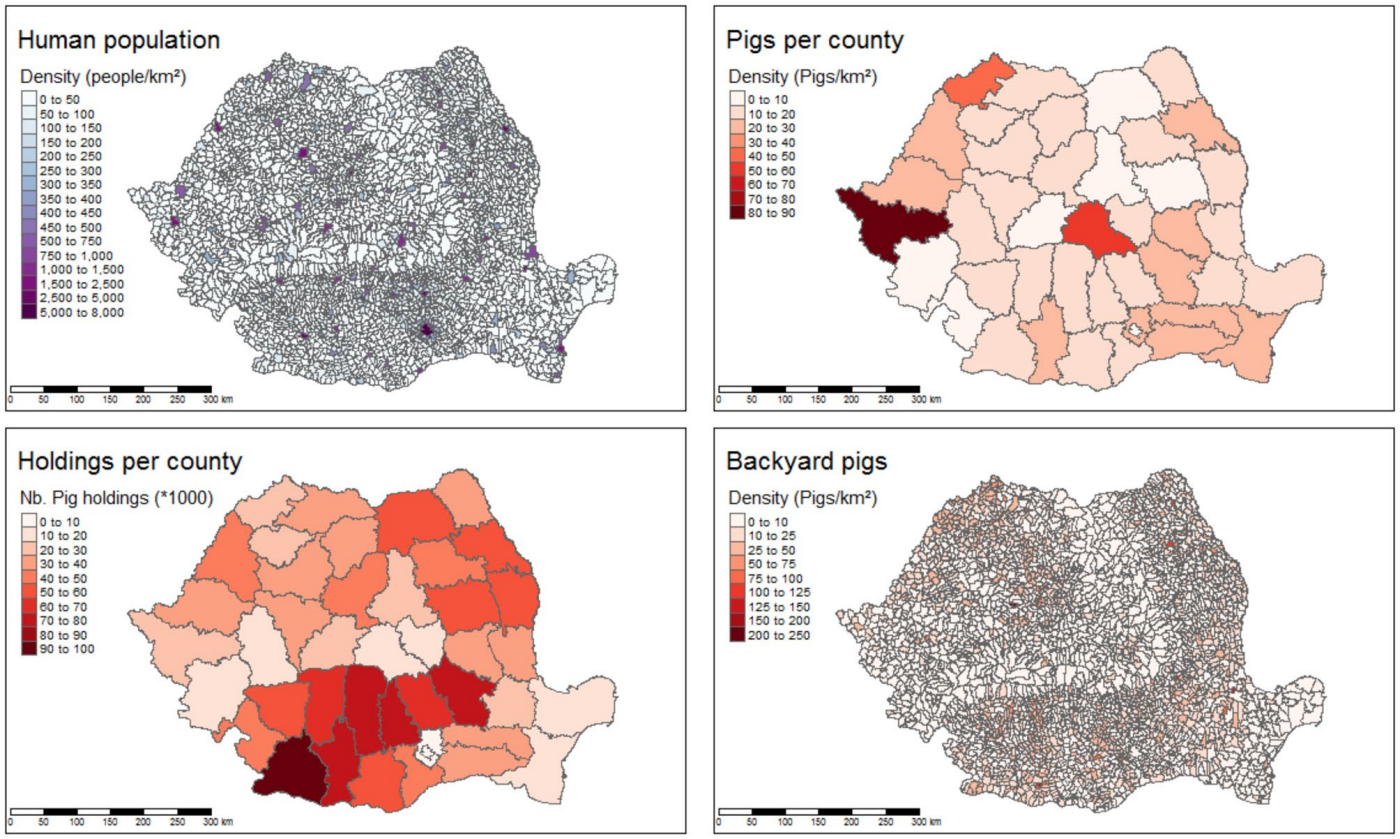
Demographic and agricultural data. Data are presented at the commune level (human population, backyard pigs) or at the county level (pigs, holdings).

Romania has approximately 5 million domestic pigs distributed in industrial sites, representing 55% of the total population, or familial swine holdings, i.e. backyard holdings representing 45%. About 700,000 animals are located in the county of Timis, mostly raised at 44 large industrial sites and accounting for 92% of the population. In contrast, the county of Dolj accounts for the largest number of sites with approximately 100,000 holdings for a total population of about 150,000 pigs (Fig. 2).

**Figure 3:**
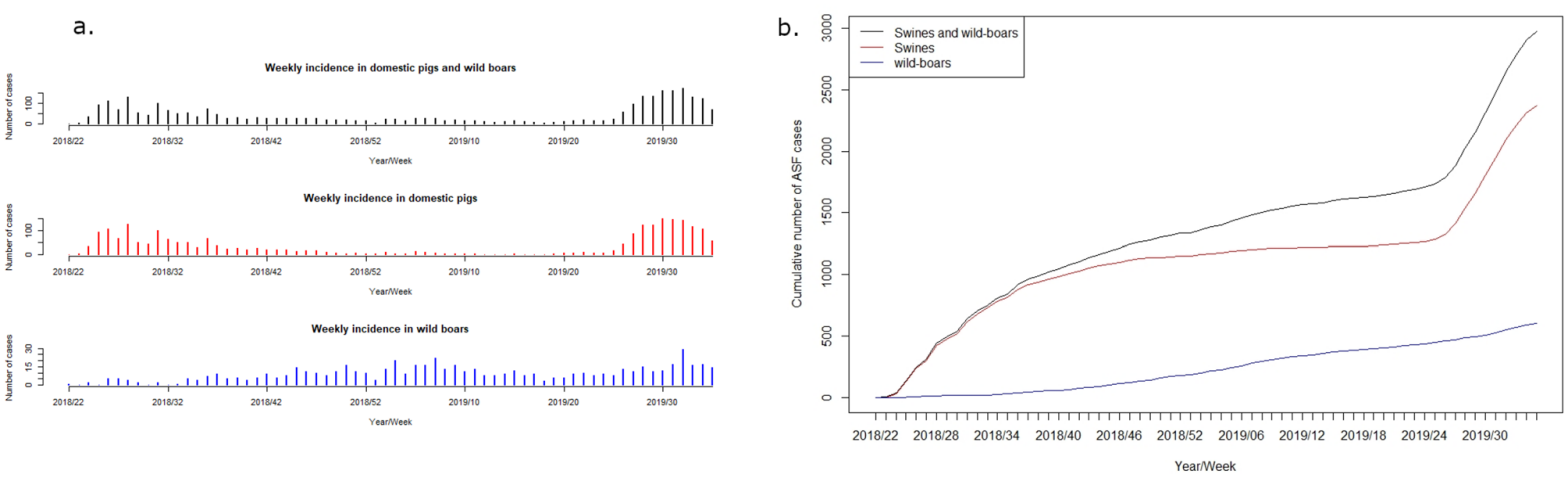
Temporal characteristics of the ASF epidemics in Romania (epidemiologic data): weekly incidence in wild boars, domestic pigs and total population (**a**), and cumulative incidence throughout the period (**b**).

The first case of ASF in Romania was officially reported in the county of Satu Mare on July 31, 2017, which was followed by six sporadic notifications (in domestic pigs and wild boars) in the same county within the next 6 months. The epidemic phase was observed 1 year later, at a distance of about 500 km, in the county of Tulcea. In contrast with the first viral incursion, these epidemics grew dramatically with up to 50 notifications within 1 week. Based on these considerations, we limited our analysis to epidemic data from the first case notification in Tulcea county, ignoring the first sporadic cases that occurred in Satu Mare. The period considered for the analysis therefore ran from June 10, 2018 to August 29, 2019. A total number of 2973 confirmed ASF outbreaks were recorded in domestic pigs and wild boars throughout this period. The temporal course of the epidemic revealed different patterns in domestic pigs and wild boars. As shown in Fig. 3a, the number of new cases among the domestic pig population exhibited two distinct peaks, in summer 2018 and summer 2019 respectively, while the cumulative number of infected wild boars increased quasi-linearly throughout the study period (Fig. 3b).

**Figure 4:**
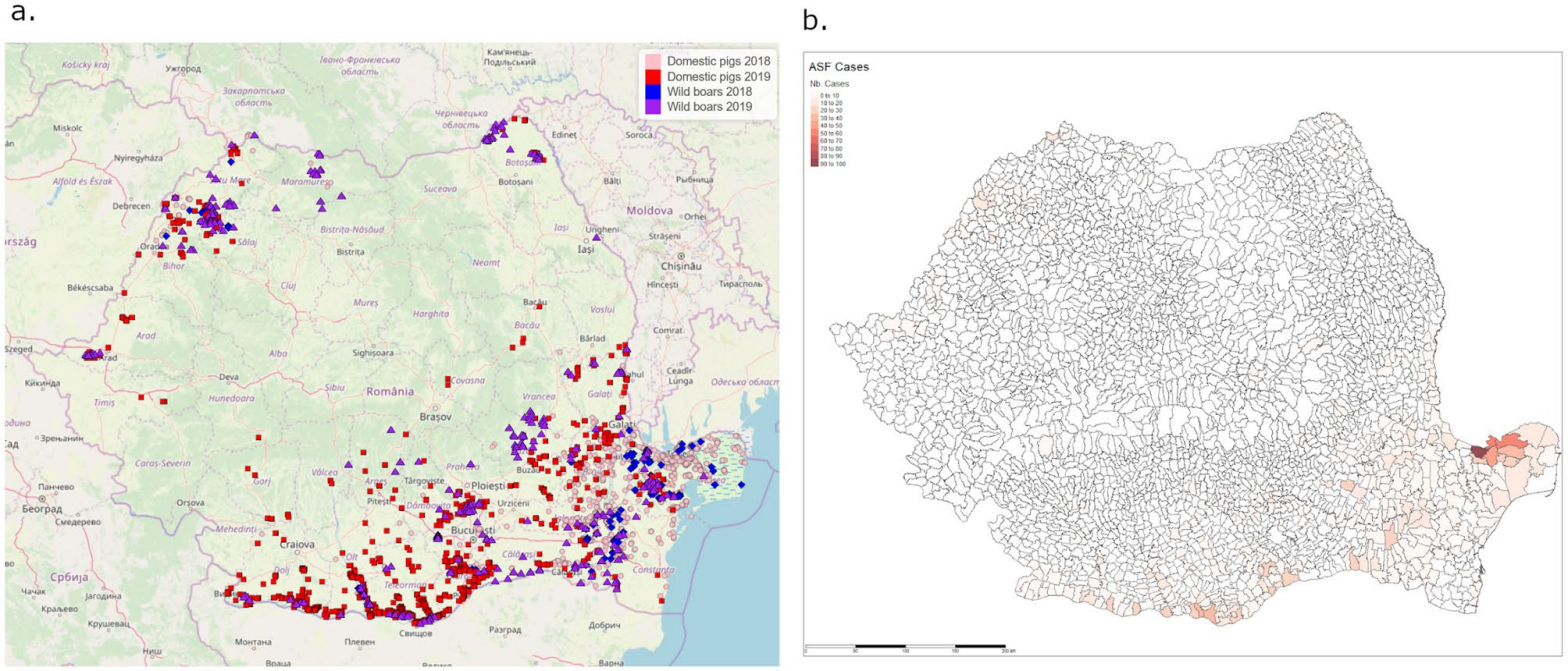
Spatial expansion of the ASF epidemics in Romania from June 10, 2018 to August 29, 2019; (**a**) case locations (map created using R^17^ (version 4.0.2), packages tmap^18^ and rgdal^19^), (**b**) number of domestic pig outbreaks per commune.

Starting in Tulcea county, the virus spread along the Danube Delta and the western coast of the Black Sea. The virus then expanded westerly to Braila and Galati counties (Fig. 4a, pink dots). By the end of 2018, 1151 domestic pig holdings were declared ASF-positive. The epidemiological situation was stable during the first semester of 2019. However, a turning point in the epidemic was observed in June 2019 with a sudden increase in the number of cases reaching 2351 on August 29, 2019 in 231 communes (Fig. 4b). This secondary outbreak mostly extended along the borders with Bulgaria and Hungary (Fig. 4a, red dots).

### Spatiotemporal clustering

**Figure 5:**
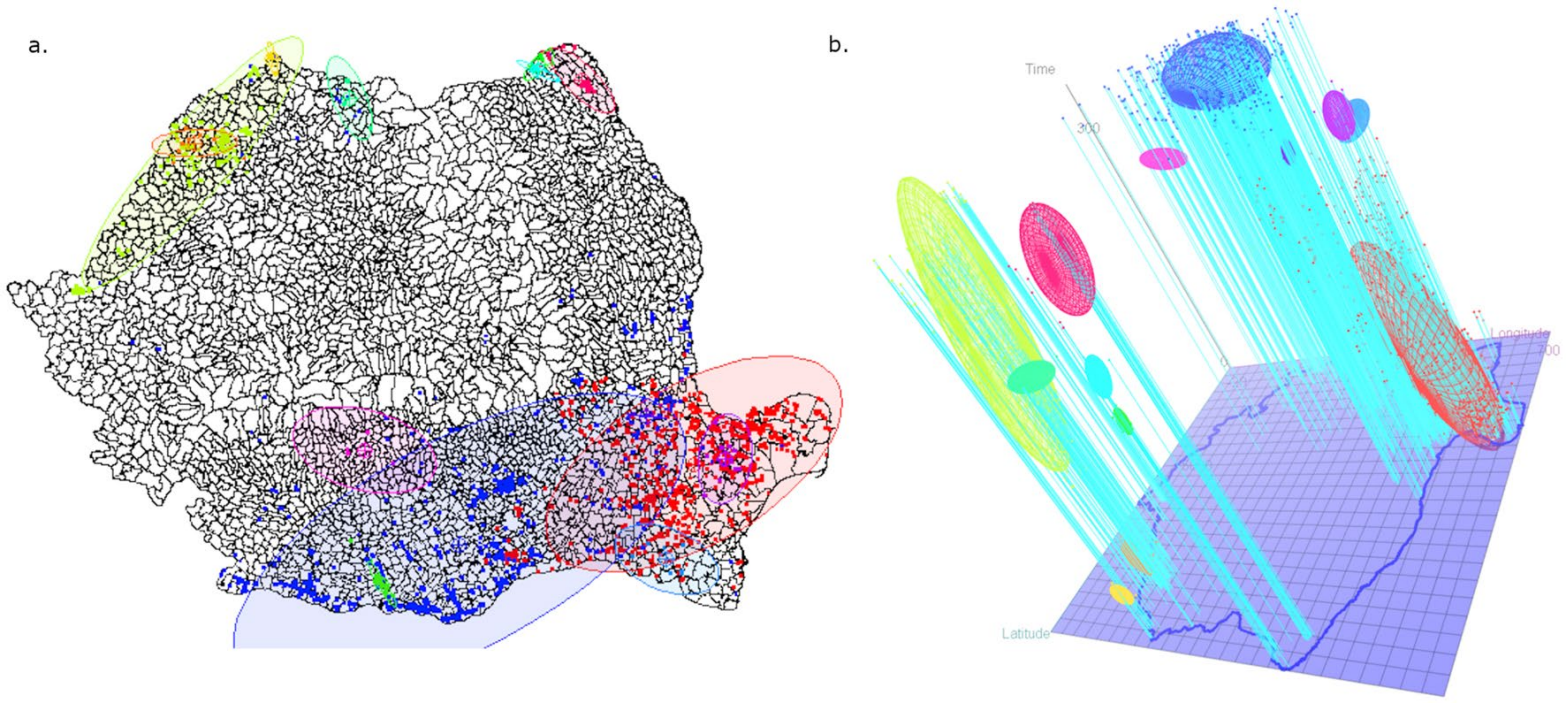
Geographic distribution of the 14 spatiotemporal clusters from June 10, 2018 to August 29, 2019 (N = 2973 cases; 2371 pig holding outbreaks and 602 wild boars cases). (**a**) 2-dimensional representation; (**b**) 3-dimensional plot, time being represented along the third (vertical) axis (interactive view available on request).

The number of cases to be classified was N = 2973, including 2371 pig holding outbreaks and 602 wild boar cases. The *eps* parameter governing the spatial distances between cases within clusters was fixed at 0.5 and concerns both longitude and latitude. With this distance, the close surroundings of each commune were likely to belong to the same cluster whenever cases were declared in the same time window. Values from 5 to 30 days were tested for the time window clustering parameter (*eps2* parameter). A 20-day period, giving an interpretable and stable number of clusters, was selected. For analyses being carried out at the commune scale, the average number of outbreaks in infected communes (5 cases) was considered as the minimum cluster size (*minpts* parameter). The ST-DBSCAN clustering algorithm identified 14 spatiotemporal clusters that can be interpreted as areas of epidemiologically related cases. The three largest clusters represented 93% of ASF cases, including 1319, 1138 and 243 cases, respectively. The size of the remaining clusters varied between 56 and 4 cases, while 78 cases were considered outliers. The geographic and temporal locations of the clusters are described in Table 1, illustrated in Fig. 5a,b, and are further detailed in chronological order.

**Table 1:**
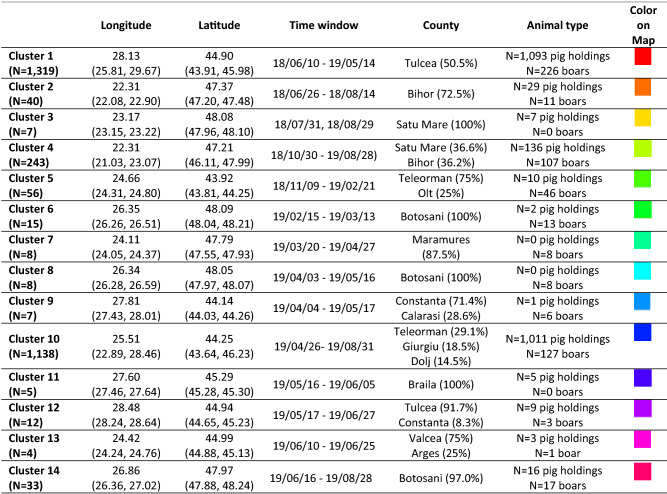
Description of the 14 spatiotemporal clusters of ASF in Romania from June 10, 2018 to August 29, 2019 (N = 2973 cases; 2371 pig holding outbreaks, 602 wild boar cases), given in chronological order.

Mean values, minimum and maximum (bracketed) values for longitudinal and latitudinal expansion. The counties in which the majority of cases for each cluster is given along with the percentage of cases in these counties. The colors correspond to the ellipsoids in Fig. 5.

Cluster 1 corresponds to the first cases that appeared at the beginning of the study period (June 10, 2018) and extended to May 2019 in the county of Tulcea (east of Romania); more than 50% of outbreaks occurred during the first 4 months after introduction. In all, 1319 sites were recorded, including 1093 domestic pig holdings and 226 wild boar carcasses. Then, in summer 2018, 40 cases (pig holdings and wild boars) were reported in the county of Bihor (Cluster 2), directly followed by the third cluster in August 2018 in the county of Satu Mare (west; 7 domestic pig holdings). Although not included in Cluster 4, the two latter clusters might be the source of the epidemics in this Cluster. In total, 243 cases (pigs and wild boars) were reported in Satu Mare and Bihor counties over a long period ranging from October 30, 2018 to August 28, 2019; most of these cases occurred in spring–summer 2019. Up to April 2019, five local outbreaks (clusters 5–9) of relatively small size, ranging from 7 to 56 cases, were detected. These clusters mainly concerned wild boars, with only 13 cases in domestic pig holdings. However, on April 26, 2019 the second epidemic wave appeared, corresponding to the largest cluster (Cluster 10) which gathered 1138 cases in the counties of Teleorman, Giurgiu and Dolj (south), 90% of which in domestic pig holdings. In May 2019, two new smaller clusters appeared in the east: Cluster 11 and Cluster 12, in the counties of Tulcea and Braila, respectively. In June 2019, the small Cluster 13 was detected in the center of Romania (Valcea and Arges counties), followed by another hotspot (Cluster 14) of 33 cases in Botosani (northeast). The spatiotemporal clustering demonstrated different spreading routes especially in the three large clusters. Clearly, the first large epidemic wave in Tulcea affected a large number of swine holdings within a spatially restricted area (Fig. 5b), covering the Danube Delta and the Black Sea coast. Despite the long duration of this outbreak (about 11 months), ASF cases remained in a relatively small area. In contrast, the southern and western outbreaks revealed wide spatial distribution in a relatively short time window (Fig. 5b).

**Figure 6:**
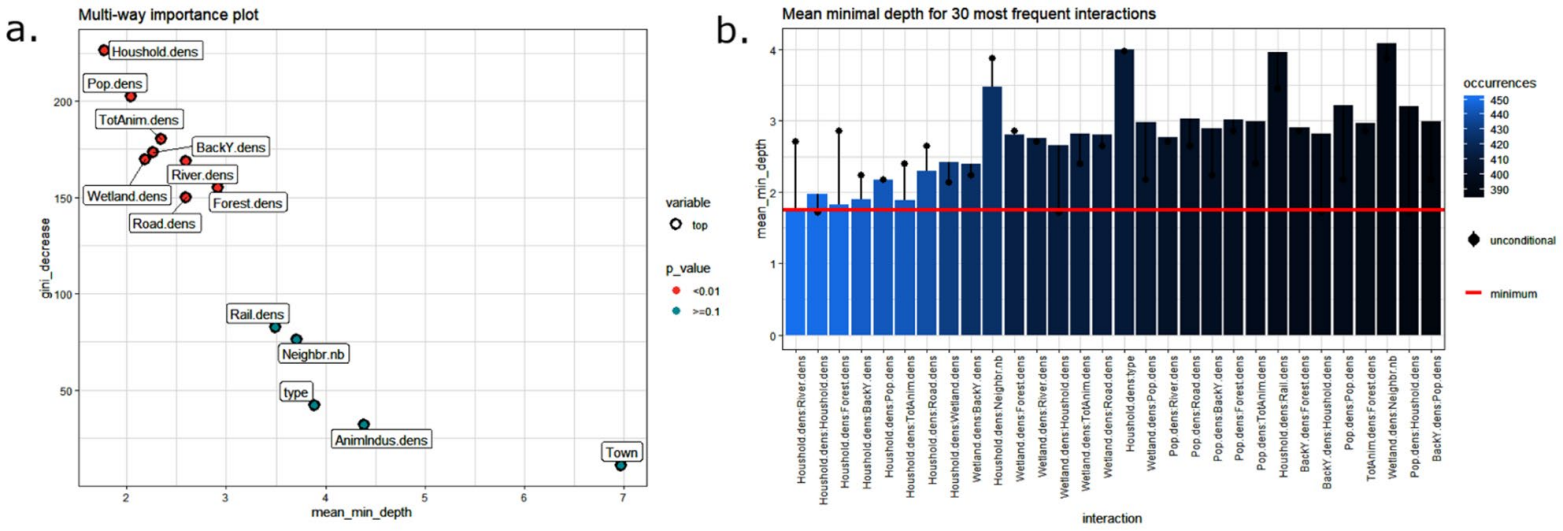
Random forest classification for the 14 spatiotemporal clusters of ASF in Romania. (**a**) Explanatory variable importance plot to differentiate clusters (N = 2895 cases, including 2322 pig holding outbreaks and 573 wild boar cases from June 10, 2018 to August 29, 2019). (**b**) Most frequent interactions between the eight significant explanatory variables explaining the 14 spatiotemporal clusters of ASF in Romania from June 10, 2018 to August 29, 2019 by means of a random forest classification. N = 2895 cases, including 2322 pig holding outbreaks and 573 wild boar cases.

In a second step, we therefore analyzed whether geographic, agricultural, and demographic variables could be used as determinants of the spatiotemporal patterns of the 14 spatiotemporal clusters (other than spatial and temporal) using a random forest algorithm. The OOB error is equal to 8.67%. Three main indexes relative to the 14 explanatory variables’ importance (i.e. Gini decrease, mean depth, and significance) were extracted and plotted in Fig. 6a.

The eight (out of 14) significant variables explaining the observed spatiotemporal clusters were household density, population density, density of total pigs and of backyard pigs, wetlands, rivers, forests, and road densities. Finally, the interactions between the significant variables are studied and illustrated in Fig. 6b. The most frequent interactions between explanatory variables (= maximum number of occurrences) are illustrated on the left part of the plot (in light blue). They are represented as decreasing number of occurrences; the first eight concern household density, with the lowest MMD (1.8). For instance, for interpretation of the main interaction, household density was strongly associated with river and forest densities, and allowed for a decrease of the unconditional MMD of the forest density variable of 1 point (2.8 to 1.8 and 2.9 to 1.9 for rivers and forests, respectively) when interacting with household density. In other words, the impact of river and forest densities is mostly important when associated with household density. Maximal subtrees of household density variables were found to split six other roots immediately: household density (in the decision tree, a variable may interact with itself when used several times to build a tree), backyard density, population density, density of total animals, and road and wetland densities. All these variables are in addition associated with the minimal MMD, meaning that these interactions have a strong impact on the prediction.

### Risk-factor analysis

Preliminary analyses considering the 2018-based model used to predict data from 2019 showed that the 2018 and 2019 ASF pig holding outbreaks were clearly associated with different risk factors. Therefore, in the following, two models of risk factors are proposed, one for the 2018 data and another for the 2019 data. Taking the 2939 communes as epidemiologic units, the number of pig holding outbreaks per commune was considered to be the dependent variable in each period (i.e. in 2018 or in 2019). Fourteen potential risk factors were measured: thirteen at the commune level and one at the county level (i.e. the number of pig holdings, Fig. 2). The thirteen commune-level variables were the densities of forests, wetlands, rivers, roads and railways; the density of population and households; the number of towns and of neighboring communes; the density of industrial pigs, of backyard pigs and of total animals, and the number of infected wild boars in each period (Figs. 1 and 2). Two random forest regressions were applied and interpreted in the following presentation of results.

#### 2018 risk factors

**Figure 7:**
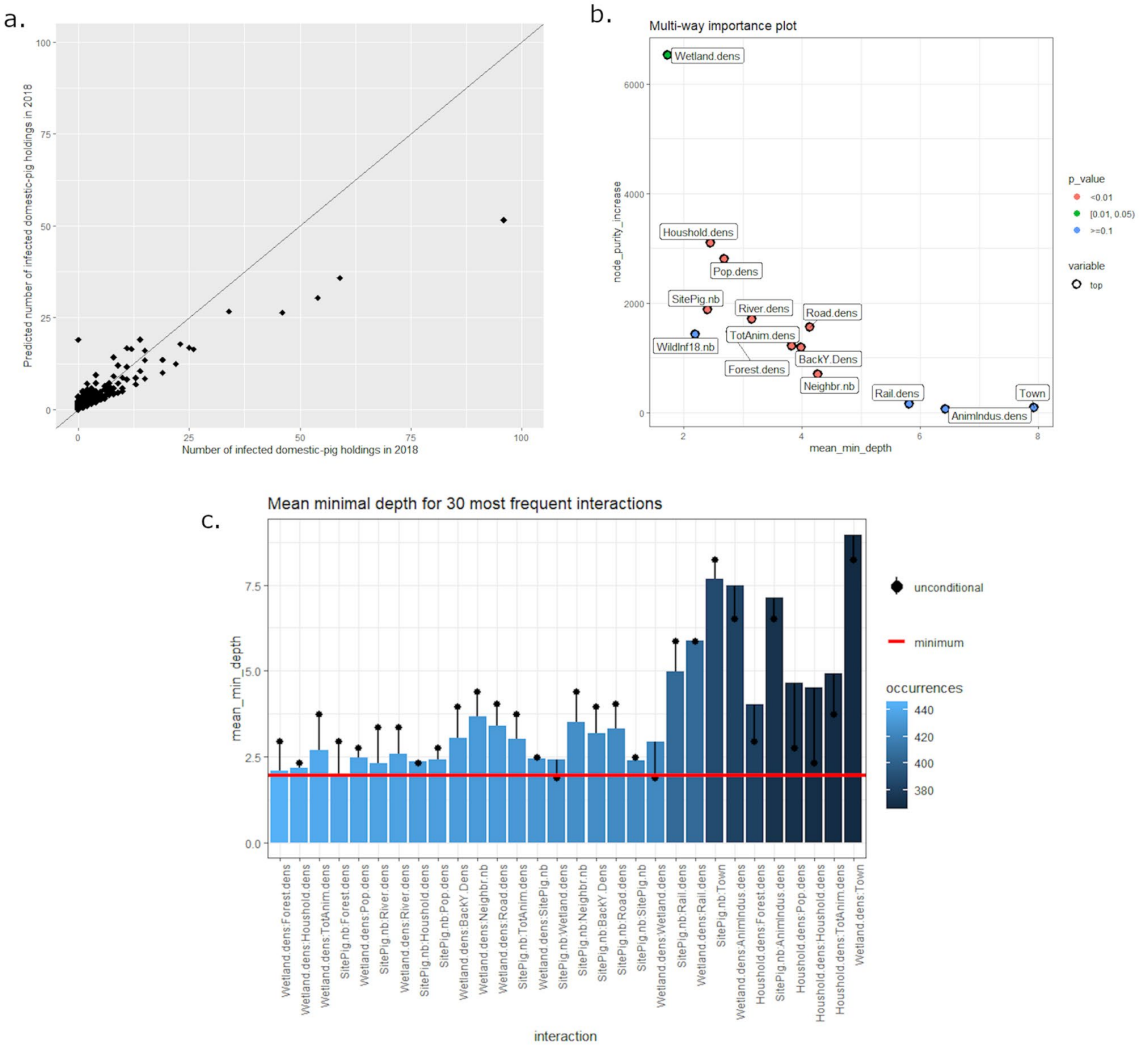
Random forest regression results for 2018 ASF outbreaks in Romania at the commune scale (N = 2939 communes). (**a**) Observed *versus* predicted values of the number ASF outbreaks (pig holdings) per commune. (**b**) Explanatory variable importance plot considering node purity increase and mean minimal depth. (**c**) Mean minimal depth for 30 most frequent interactions between the 10 significant explanatory variables.

The mean of squared residuals was equal to 7.27 and the percentage of explained variance was equal to 19.2%, which allows us to consider the model to be of high quality (Fig. 7a). Three main indexes relative to the 14 explanatory variables’ importance (i.e. node purity increase, mean depth, and significance) were extracted and plotted in Fig. 7b. The number of infected domestic pig holdings in 2018 was mainly related to 10 explanatory variables: wetland density, household density, population density, number of pig sites, river density, road density, forest density, total pig density, backyard density, and number of neighboring communes. In addition, the interactions between the significant variables were studied and illustrated in Fig. 7c. The most frequent interactions between explanatory variables (= maximum number of occurrences) are illustrated on the left part of the plot (in light blue). The first seven interactions involved environmental variables (wetland, forest and river densities). Importantly, these variables are also associated with population variables. These interactions are usually associated with low MMD values, meaning that they have a strong impact on the prediction. Most of these interactions allow for a decrease of the unconditional MMD of the root variables, when interacting variables are considered.

#### 2019 risk factors

**Figure 8:**
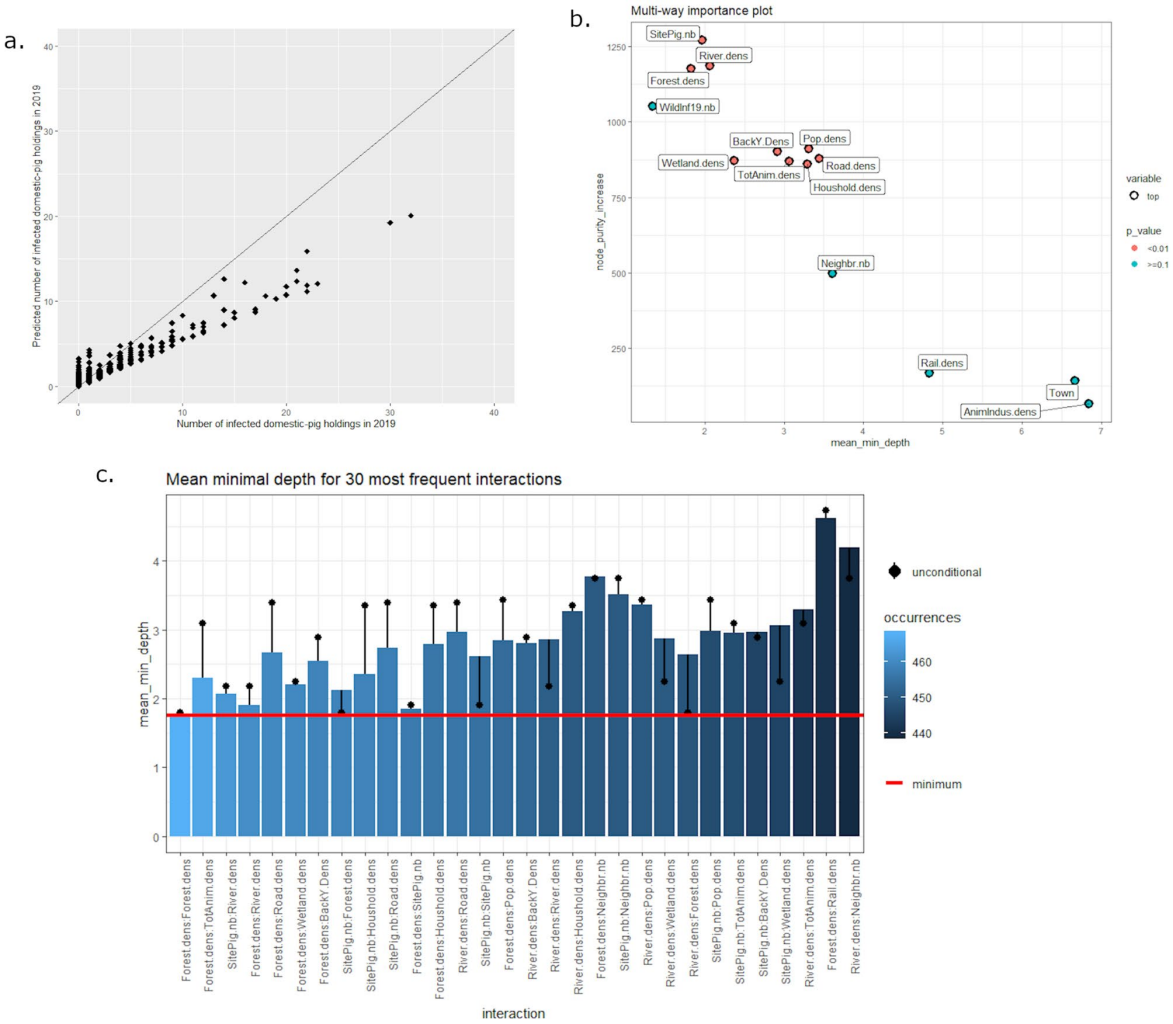
Random forest regression results for 2019 ASF outbreaks in Romania at the commune scale (N = 2939 communes). (**a**) Observed *versus* predicted values of the number ASF outbreaks (pig holdings) per commune. (**b**) Explanatory variable importance plot considering node purity increase and mean minimal depth. (**c**) Mean minimal depth for 30 most frequent interactions between the 10 significant explanatory variables.

The mean of squared residuals was equal to 3.33 and the percentage of explained variance was equal to 20.4%. The model quality was evaluated with the root mean square of calibration, illustrated in Fig. 8a. Concerning these data, model quality was considered to be good. Three main indexes relative to the 14 explanatory variables’ importance (i.e. node purity increase, mean depth, and significance) were extracted and plotted in Fig. 8b. The number of infected domestic pig holdings per commune in 2019 was mainly influenced by nine explanatory variables: number of pig sites, river density, forest density, population density, total animal and backyard pig density, wetland, road, and household densities. In addition, the interactions between the significant variables are studied and illustrated in Fig. 8c. The most frequent interactions (maximum number of occurrences) are illustrated on the left part of the plot (in light blue). The most representative interactions involved the forest density variable, coupled with animal population variables (number of pig sites and animal density). River densities were also identified as important interacting variables, resulting in the highest decrease of MMD when interaction with the forest density variable is considered. These interactions are usually associated with low values of MMD, meaning that they have a strong impact on the prediction.

## Discussion and conclusion

African swine fever is one of the most important diseases affecting pigs and was introduced into the European continent 12 years ago. It has spread rapidly to many countries that were ASF-free for decades. Romania has been very strongly affected in the last 2 years (2018 and 2019). The first cases were reported from July 2017, and since then, about 3000 cases have been confirmed up to mid-2019 among domestic pigs and wild boars. During this 2-year period, two epidemic phases can easily be identified, with two peaks of case notifications separated by in-between low-noise spread. The Animal Diseases Notification System database provides temporal and geolocalization data for each case, enabling spatiotemporal analysis. Three large clusters, gathering 80% of cases, could be identified. Two of these clusters were strongly associated with the two epidemic waves, but revealed different spreading patterns. Interestingly, the first one in 2018 extended over a large time-period, but within a relatively restricted area (Tulcea county and the Danube Delta). In contrast, the second cluster, covering the 2019 epidemics, occurred on a short time scale, but with broad geographic expansion from south-west to south-east. Finally, the third cluster, although of relatively small size, covered a large western part of the country and lasted throughout the whole study period.

Alongside the temporal characteristics, the role of external factors on the cluster patterns was assessed using the random forest algorithm. Human and animal demographic data, and geographic and agricultural characteristics, were collected and considered to be potential explanatory variables. On the one hand, a wide diversity of landscapes and land cover are present in Romania, raising the question of whether these could be drivers of the observed transmission of ASF. On the other, conversely to many countries where pig production is mostly industrialized, backyard pig production is extremely popular in Romania and strongly linked to social organization, making human activity and demography focal points for transmission of the virus within its target population. This was confirmed using the modelling approach, which enabled us to highlight household density as the main determinant of cluster membership, along with pig population characteristics (e.g. number of backyard animals), followed by environmental variables, in particular wetland and forest cover. Forest cover was found to be of primary importance for cluster membership attribution when associated with the household density variable. Altogether, this suggests that related outbreaks (i.e. belonging to the same cluster) were driven by a triad, including (i) human activity (population density, household density, roads), (ii) backyard holding population, and (iii) environmental characteristics (forests, rivers and wetlands).

The second step of the study focused on the characteristics of ASF outbreaks at the local scale, taking the commune as the epidemiologic unit. Owing to their specificities, the two epidemic waves were analyzed separately to assess the relevant and specific demographic and environmental determinants of the outbreaks. For both outbreaks, environmental data were found to be the main predictors of the outbreak size at the commune level, with wetland and forest cover for the years 2018 and 2019, respectively. Along with wetlands in 2018, rivers were also identified as a pivotal factor in 2019, highlighting water as a key driving force for the infection. The cases were principally located in the Danube Delta and along the Danube on the border with Bulgaria. This raises the question of water supply to backyard animals and potential contamination of waterways by wild boar carcasses or waste. However, the analysis of interactions clearly showed that river density was strongly correlated with the transportation network (roads), the distribution of the human population, together with the pig population. Therefore, we cannot exclude that rivers may constitute a confounder of other factors more related to human activity and the distribution of the pig population. As a result, the human structure again appears determinant, especially household density. Due to the sociologic and economic conditions in Romania, this variable could reflect the number of backyard holdings and therefore be a good approximation of the targeted susceptible population.

The major role of anthropogenic factors represented by the density of roads, water bodies, and the domestic swine population (mainly backyard pigs) was also found in a similar study in the Russian Federation^16^. Similarly to our results, the study found an unexpected high influence of the density of water bodies, and the authors assumed this factor was strongly related to the habitat of wild boars. Even though we cannot exclude this explanation, the interactions with other variables more related to human activity found in our study suggest predominant involvement of human activity closely linked to the geographic features of the country.

The role of wild boars in domestic pig infection is likely in some cases because of the low level of biosecurity in backwards holdings; however, we did not identify a clear link between identified wild boar cases and domestic pig outbreaks, especially in 2018. However, this lack of relationship may be due to the relatively low reporting of wild boar cases during this period. In fact, the number of cases in the wild population was highlighted as an explanatory variable only for the 2019 outbreak. It is clear from the data that the number of reported cases in wild boars was higher during this second period, but we could consider whether this difference is real or due to improved detection of carcasses. In the Russian Federation, the driving force behind the epidemic in its preliminary stages was direct contact of infected wild boars between each other and with traditionally free-ranging domestic pigs on backyard farms. However, the next stage developed mainly because of human activity—illegal movement of contaminated pig products from affected regions and swill feeding^20^.

A similar pattern was also observed more recently in the epidemics that have affected China^21^. The fact that for the 2018 Romanian epidemics, wetland was found to be the main driver and then in 2019, the explanation moved to greater involvement of anthropogenic variables such as the distribution of pig holdings, roads and rivers, also suggests a change in the factors involved. In the initial introduction of the disease, we can assume a strong interaction between domestic pigs and wild boars in the highly specific area of the Danube Delta, and then increased disease spread due to human activity.

The Romanian ASF situation is unique, due to both the duration of the outbreak and the number of outbreaks actually reported by the authorities. Although the epidemiologic context is probably closely related to the way pig production is organized in the country, lessons should be learned from this experience. Despite strong intervention policies and public awareness initiatives, the ASF virus continued to spread in the wild and domestic population, reaching about 3000 holdings. Land cover and water were identified as pivotal factors, but they cannot hide the role of human activity, which was identified throughout our study, both at the national and local scales. Transposing to intensive farming, these findings highlight the need for strict biosecurity measures both on farms and during transportation.

## Materials and methods

### Data

Four complementary data sources were used to analyze the spatiotemporal patterns of ASF spread, and to identify the risk factors associated with the spread of the disease in Romania: geographic, demographic, agricultural, and epidemiologic data.

#### Geographic data

Geospatial data were extracted from a publicly available European-level database called EuroGlobalMap (EGM), administered by Eurogeographics. With a resolution of 1:1,000,000, this database covers 46 European countries and provides hydrologic, transportation, and landscape data. Data for Romania were extracted and analyzed at the administrative scales corresponding to communes and counties. Layers corresponding to hydrologic data (waterways) and landscape (wetlands, forests and urban areas) were considered. Human activity was also taken into account through transportation infrastructures (roads and railways).

#### Demographic data

The National Institute of Statistics of Romania (http://www.insse.ro) provides a wide range of datasets regarding demographic characteristics and urbanization. From these datasets, we selected those thought to be relevant concerning the spread of ASF. Importantly, the distribution of domestic pigs is considered to be strongly related to human occupancy, especially in low-urbanized areas. Therefore, the population density, the predominance of urban areas, and the number of households were accounted for in the analysis.

#### Agricultural data

Data on agricultural activity were focused exclusively on domestic pigs and were provided by the Romanian authorities (ANSVSA) or extracted from the county Sanitary Veterinary and Food Safety Direction websites (DSVSA). These data consist in a census of live pigs at the commune level, as well as the number of industrial holdings, and their capacities. We were therefore able to determine the number of backyard animals in each locality. The total number of domestic pig holdings per county was obtained from the 2010 general agricultural census carried out by the National Institute of Statistics of Romania (http://www.insse.ro/cms/files/RGA2010/Rezultate%20definitive%20RGA%202010/rezultate%20definitive%20RGA%202010.htm).

#### Epidemiologic data

The Animal Diseases Notification System (ADNS) registers and documents changes in the epidemiologic context in Europe, providing accurate data on the location and characteristics of infected sites and species—wild boars and domestic pigs for ASF—using GPS coordinates. The epidemiologic unit for domestic pigs is the holding, whereas it is the individual for wild boars.

### Methods

#### Spatiotemporal clustering: identifying homogeneous hotspots and related risk factors

Our first objective was to detect clusters by analyzing the spatial closeness and temporal similarities of the confirmed ASF outbreaks, i.e. domestic pig outbreaks and wild boar cases. A spatiotemporal clustering method was applied to build clusters of cases. In accordance with the format of our data, a density-based method (e.g., ST-DBSCAN) was preferred over a space–time scanning method (e.g., SaTScan). The density-based method has the advantage of being able to take into account a large number of cases, and to detect irregularly shaped clusters^22^. Density-based methods define clusters as areas of high densities of cases—interpreted as hotspots—separated by areas of low densities.

##### Clustering algorithm

The ST-DBSCAN clustering algorithm was applied^23^. It extends the well-known DBSCAN algorithm focused only on spatial data. The ST-DBSCAN algorithm is based on density calculated with two-distance radii: a spatial radius and another, which can be temporal for instance. The density estimate is the number of cases within a neighborhood. Euclidean distance is adopted to compute neighborhood distances. Three parameters must be filled in: (i) “eps”, the neighborhood radius considered for density estimation of spatial attributes (i.e. latitude and longitude), (ii) “eps2”, the neighborhood radius considered for density estimation of temporal attributes, and (iii) “minpts”, the density threshold (i.e. minimum number of cases) used to detect dense areas, and to classify cases according to their status (i.e. core/border/outlier)^23,24^. The stdbscan R function was used (https://github.com/Kersauson/ST-DBSCAN).

##### Cluster interpretation

The aim was to highlight explanatory variables that best differentiate the spatiotemporal cluster memberships. A classification method was applied where the categorical dependent variable was the cluster membership, and the explanatory variables the, geographic, demographic, agricultural, and epidemiologic data (described in “Data description” section). It is important to mention that these explanatory variables are likely to have strong correlations. Therefore, a non-parametric classification method, such as a classification tree^25^, was selected. At the time of the development of machine learning, the random forest algorithm^26^ was preferred to a classification tree because it has the advantage of correcting overfitting, and thus delivers robust results with exceptional empirical accuracy.

#### Risk-factors: assessing at the local scale

Our second objective was to identify risk factors for domestic pig infection related to the spatial risk of ASF at the commune level. The model aimed at modelling the number of ASF outbreaks detected in domestic pigs at the commune scale considering geospatial, demographic, and agricultural data (described in “Data description” section) as explanatory variables. To obtain a robust and strongly explanatory model, even with highly correlated explanatory variables, the random forest algorithm was also applied as a non-parametric regression method better defined as a supervised machine learning algorithm^26^.

#### Random forest methodology

The random forest algorithm^26^ is an ensemble learning method that operates by constructing a collection of decision trees^25^. A decision tree is a non-parametric statistical analysis that can be applied to classification (i.e. a categorical dependent variable) or to regression (i.e. a quantitative dependent variable). A decision tree is built on the whole dataset, whereas a random forest randomly selects observations and variables to build a large number of decision trees and then averages the results. More precisely, the set of decision trees in a random forest (500 trees) results from two sources of randomness: (i) initial observations are modified by BAGGING (= Bootstrap and AGGregatING), and (ii) initial variables are modified by a random selection of P' variables among P (usually set to ${P}^{^{\prime}}=\sqrt{P}$ for classification and *P'* = *P/3* for regression). The final random forest model is selected from this ensemble of decision trees by a majority vote, i.e. it aggregates the votes from all the trees to decide on the final class of the observation.

##### Interpretation tools

The random forest algorithm is associated with four interpretation aids. Based on the high number of observations and the relatively low number of parameters, Out-Of-Bag (OOB) and Cross-validation (CV) error would behave similarly^27^. Owing to this, OOB being a classical indicator for RF, we used OOB for evaluating the quality of model outcomes. First, the overall quality of the final model is evaluated by the Out-Of-Bag (OOB) error, processed on all the bootstrapped trees that do not contain observations used to process the OOB error. This error is a percentage of correctly classified observations (classification) or a mean of squared residuals (regression). Second, the importance of an explanatory variable in the model derives from the OOB error^28^. For the purposes of classification, the importance corresponds to the mean decrease in the Gini index of node impurity by splits on the variable under study (i.e. values for this variable are randomly swapped in OOB samples). Of note, swapping the rows of a “noise” variable tends neither to increase nor to decrease node purities, while swapping the rows of an “important” variable tends to yield a relatively large decrease in the Gini index. For the purposes of regression, the importance of the variable is measured by the node purity increase index. Third, two additional indexes relative to this importance can be given: the mean minimal depth (MMD) and the significance of the variable’s importance. The MMD assumes that variables with a strong impact on the prediction are those that more frequently split nodes nearest to the trunks of the trees (i.e. at the root node). This index is measured by averaging the depth of the first split for each variable over all the trees within the forest. Lower values of this measure indicate variables that are important in splitting observations. The significance is based on a binomial test that tells us whether the observed number of successes (i.e. number of nodes in which the variable was used for splitting) exceeds the theoretical number of successes if they were at random. Fourth, the most frequent interactions (occurrences) between explanatory variables (i.e. splits appearing in maximal subtrees with respect to one of the variables selected) can be given. In addition, these occurrences can be associated with the MMD of these interactions.

##### In practice

The RandomForest and the randomForestExplainer R packages were used (https://github.com/ModelOriented/randomForestExplainer). To get reproducible results, the arguments of the functions of these two packages are set as the default ones.
